# *Mangifera sylvatica* (Wild Mango): A new cocoa butter alternative

**DOI:** 10.1038/srep32050

**Published:** 2016-08-24

**Authors:** Sayma Akhter, Morag A. McDonald, Ray Marriott

**Affiliations:** 1 School of Environment, Natural Resources and Geography, Bangor University, Gwynedd, LL57 2UW, UK; 2 Biocomposites Centre, Bangor University, Gwynedd, LL57 2UW, UK

## Abstract

Cocoa butter is the pure butter extracted from cocoa beans and is a major ingredient in the chocolate industry. Global production of cocoa is in decline due to crop failure, diseases and ageing plantations, leading to price fluctuations and the necessity for the industry to find high quality cocoa butter alternatives. This study explored the potential of a wild mango (*Mangifera sylvatica*), an underutilised fruit in south-east Asia, as a new Cocoa Butter Alternative (CBA). Analyses showed that wild mango butter has a light coloured fat with a similar fatty acid profile (palmitic, stearic and oleic acid) and triglyceride profile (POP, SOS and POS) to cocoa butter. Thermal and physical properties are also similar to cocoa butter. Additionally, wild mango butter comprises 65% SOS (1, 3-distearoyl-2-oleoyl-glycerol) which indicates potential to become a Cocoa Butter Improver (an enhancement of CBA). It is concluded that these attractive properties of wild mango could be prompted by a coalition of policy makers, foresters, food industries and horticulturists to promote more widespread cultivation of this wild fruit species to realise the market opportunity.

Cocoa butter (CB) is a light yellow fat obtained from beans of the cocoa plant (*Theobroma cacao* L.). It is one of the unique natural fats highly demanded by food, pharmaceuticals and cosmetic industries^1^,^2^. Cocoa butter is the major ingredient of the chocolate industry^3^. Palmitic acid (C16:0), stearic acid (C18:0), oleic acid (C18:1) and linoleic acid (C18:2) account for more than 98% of the total fatty acids^4^ in cocoa butter. This is the only commercially available natural fat which is rich in saturated and monounsaturated fatty acids,13.6–15.5% of 1,3-dipalmitoyl-2-oleoyl-glycerol (POP), 33.7–40.5% of 1-palmitoyl-3- stearoyl-2-oleoyl-glycerol (POS) and 23.8–31.2% of 1,3-distearoyl-2-oleoyl-glycerol (SOS)^5^,^6^. These relatively simple triglycerides in cocoa butter confer its desirable melting profiles prized by the confectionery industry, being solid at 20 °C and melting between 27–35 °C which is appreciated by consumers as well as desirable in confectionery applications^7^. Moreover, the price of cocoa butter is one of the highest among all tropical fats and oils^5^,^7^,^8^. According to ICCO (2015), the price of cocoa butter more than doubled between 2005 and 2015^9^, from $1433/tonne to$3360/tonne (Supplementary Fig. 1). Cocoa is cultivated on a land area of over 70,000 km^2^ worldwide^10^ while Africa (68%), Asia (17%) and America (15%) contribute the major proportion of global production of CB^10^. According to ICCO^9^, annual global cocoa production was reported to be more than 4 million tonnes per season. However, global demand for cocoa is growing annually by 2 to 3% due to low productivity, price fluctuations and uncertainty in supply (Supplementary Fig. 1), which has forced the confectionery industry to seek CBAs^2^,^5^ from other natural sources^11^. Cocoa butter equivalents (CBEs) are commercially available fats containing a similar mixture of triacylglycerol to cocoa butter that can be mixed with cocoa butter up to 5%^12^. Very few tropical fats are considered to beCBAs but include those sourced from illipe butter, kokum butter, shea butter and mango (*Mangifera indica* L.) butter^13^. The mango kernel contains about 7–15% fat that is rich in palmitic, stearic and oleic acids^11^,^13^. Cocoa butter from the domesticated mango species, *M. indica*, is a natural fat containing high saturated and monounsaturated fatty acids containing symmetrical triglycerides such as POS (10 to 16%), SOS (25 to 59%) and POP (1 to 8.9%)^13^. These are relatively simple triglyceride combinations which are desirable for confectionery applications, especially in chocolate processing. *M. indica* kernels have therefore been heavily researched for their potential as a cocoa butter alternative^14^.

Wild mango (*Mangifera sylvatica* Roxb.) belongs to the Anacardiaceae family. It is found in Bangladesh, India, China, Cambodia, Myanmar, Nepal and Thailand (Fig. 1)^15^. It is one of the genetically closest species to *M. indica* in the world^16^ but it underutilized and unmarketed in its native provenance Bangladesh as well as in other tropical countries due to a lack of information and awareness of it’s potential value as a source of food, nutrition or medicine. Perhaps counterintuitively given its underutilisation, the species is already threatened in Bangladesh^17^ due to habitat loss and deforestation, but is not afforded any conservation protection due to its lack of documented value. The seed germination rate and early growth of seedlings indicates that this species could be easily domesticated and incorporated into small-scale forestry programs^15^. There is growing evidence of beneficial medicinal properties, such as a recent study showing that *M. sylvatica* leaves possess thrombolytic properties that could lyse blood clots^18^. The leaves can also be used as antidiarrheal drugs^19^. Until now, no research has been conducted on its market potential which will ultimately promote domestication and commercialisation of the species. Therefore, the present study constitutes an assessment of the potential of *M. sylvatica* as a CBA for the food, pharmaceutical and cosmetic industries. In this study, the fatty acid and triglyceride composition, and the physiochemical and thermal properties of *M. sylvatica* were determined and compared to those of the domesticated mango, and cocoa butter to assess the potential for *M. sylvatica* as a new source of cocoa butter.

## Results

### Fatty acid Profile and Triglyceride compositions

Wild mango butter (WMB) is a light yellow fat that is not greasy to touch and has a characteristic nutty flavour. WMB is a rich source of saturated fatty acids (Fig. 2a). WMB consist of three major fatty acids, namely palmitic acid (C16:0), stearic acid (C18:0) and oleic acid (C18:1) (Fig. 2b). The saturated fatty acid content of *M. sylvatica* (56%) approximates that of DMB (57%) but is lower compared to CB (65%). Stearic acid, oleic acid and palmitic acid account for 95% total fatty acid in WMB from *M. Sylvatica* followed by CB (96–97%) and DMB (94%). Apart from that, *M. sylvatica* butter contains small amounts of arachidic acid and linoleic (also known as Omega- 3 Fatty Acid), which is similar to CB and DMB (Fig. 2b). Triglycerides are complex mixtures of a variety of fatty acids, which are the major constituents in fats and oils. The major triglycerides found in WMB are 1, 3-distearoyl-2-oleoyl-glycerol (SOS), 1-palmitoyl-2-oleoyl-3-stearoyl-glycerol (POS) and 1, 3-dipalmitoyl-2-oleoyl-glycerol (POP) which is also the main features of cocoa butter (Table 1). POP, POS and SOS account for 79% for WMB and 82–85% for CB (Fig. 3h). This similarity in fatty acid and triglyceride profiles indicates considerable potential for WMB to be used as a source of cocoa butter alternative.

### Physical and thermal properties of WMB

The saponification value, glycerol percentage, iodine value, free fatty acid percentage, moisture content, specific gravity and refractive index were determined for the wild mango butters as important parameters of butter quality. In WMB the saponification value is slightly lower than CB (Fig. 3a) which means WMB consists of long chain carbon molecules but is close to DMB. WMB contains bigger carbon chain molecules so it has fewer glycerol molecules as indicated by glycerol percentage (Fig. 3b). The iodine value of WMB is slightly higher than CB (Fig. 3d) but close to DMB. An elevated iodine value and acid value indicates high susceptibility of fat to oxidative rancidity due to the high degree of unsaturation. Moisture content and free fatty acid content in WMB was high compared to other butter samples (Fig. 3c,g) which also indicate the possibility of WMB oxidation. These results suggest that proper and controlled processing can produce high quality butter with decreased degradation. The refractive index and Specific gravity of WMB butter was very similar to CB and DMB (Fig. 3f,g). This indicates the double bond present in WMB is similar like CB and the weight of WMB is very similar to CB. WMB has a melting point close to CB though DMB has a higher melting point (Fig. 4). WMB is characterised by one leading peak around 16.18 °C with a “shoulder” around −4.71 °C. *M. sylvatica* is similar to CB (Table 2) where the main melting peak appeared around 20 °C. A complete melting of WMB was observed around 26 °C and 27 °C for CB. The results from *M. sylvatica* are very different from *M. indica* where the main melting peak was observed around 16.88 °C but with multiple shoulders and with a very high melting point observed around 53 °C (Fig. 4).

## Discussion

The majority of studies report palmitic acid, stearic acid, oleic acid and linoleic acid to be the major fatty acid components of CB^20^. Minor components of lauric acid (C 12:0), myristic acid (C 14:0), linolenic (C 18:3) and arachidic acid (C 20:0) have also been reported^20^. The main difference between CB and WMB observed in this study was in the palmitic acid content, 27% and 6% respectively (Supplementary Table 2). Other studies of triglyceride content of DMB have reported highly variable results; POP (6–16%), SOS (2–59%) and POS (1–74%) and POO, SOO, SOA, OOO^7^,^13^ compared to CB which has more consistent concentrations of POS (37–47%), SOS (26–33%) and POP (16–23%) (13, 20). TGAs in WMB are similar to CB, where POP, POS and SOS are the major TGAs but with a higher percentage of SOS (65%). WMB contains a slightly lower SFA content compared to CB but the fatty acid profile is comparable (Fig. 2a,b). Therefore, it is evident that the fatty acid and triglyceride composition of WMB is close to that of CB derived from *Theobroma cacao*, indicating good prospects for WMB to be a source of cocoa butter alternative.

Key parameters in conferring high fat quality, distinctive flavour and aroma in butters are the saponification and acid values. WMB has a lower saponification value than CB (2, 21) which means the fatty acids in WMB are significantly longer carbon chain compounds (Fig. 5a). Such long chain fatty acids (saturated and unsaturated) are prone to oxidation and breakdown which provides characteristic flavours and aromas High acid values indicate breakdown of triglycerides into free fatty acids (FFA) relating to inadequate processing and storage conditions. Cocoa butter is reported to have acid values in the range of 0.42 to 3.11%^21^. The acid value of fat extracted from DMB varies from 1.22 to 7.48^7^,^22^. Our analyses showed that WMB has a significantly higher acid value compared to CB which suggests there might be processing or storage problems. With respect to iodine values, the higher the value the more reactive, less stable, softer the fat and hence more susceptible to oxidation and rancidification^7^. In general, the iodine value for CB was found to be 34–38 g I_2_/100 g^8^,^23^,^24^ and for DMB 40–75 g I_2_/100 g^7^,^22^. The iodine value of WMB fat is higher than that of CB. This study, again suggesting that adequate storage will be essential if it is to be used more widely. The moisture content of WMB is higher than CB which may render more susceptible to microbial attack and oxidation. However, the moisture content is easily managed during the extraction process. On the positive side, there is a growing body of evidence that higher moisture content butters produce more low fat chocolate which may help to prevent obesity, heart diseases, diabetics, stroke and arthritis^23^. Indeed, there is on-going research to produce low fat chocolate by adding water into the CB^24^. Manipulation of the extraction process to best manage moisture levels will eliminate the need to add water to the final product. The refractive index and specific gravity of WMB was very similar to CB. It has been suggested that the physicochemical characteristics of WMB can be manipulated through controlled processing, chemical or physical refining and natural blending processes to adjust the properties of WMB to CB^14^,^25^. The melting point is important to determine the storage temperature. The melting temperature of CB is slightly higher than WMB which could be due to of the higher saturated fatty acid content (Fig. 2a) as previously noted^23^. Similar results have been reported for CB by many researchers^2^,^14^,^26^,^27^,^28^. So, there were some significant similarities in the physical and thermal properties of WMB compared to CB which again shows the potential of WMB to be used as a cocoa butter alternative.

Chocolate commands an enviable position among food products due to its premium cost, taste and unique physicochemical properties^20^. The consumption of chocolate products has significantly increased worldwide^29^ whilst 30% of the world’s cocoa crops have been destroyed by pests and disease and are deteriorating due to climate change and ageing plantations. Demand is increasing and supply is inadequate as cocoa is cultivated in only a few tropical countries, making its availability unstable, expensive and subject to price fluctuations^20^, (Supplementary Fig. 1). Moreover, poor quality harvests and some technological problems such as fat blooms and high tempering times during chocolate production make it necessary for the food industries to look for alternatives to CB and intensive efforts are ongoing to find suitable cocoa butter alternatives^29^. Cocoa butter alternatives are divided into three subgroups (Supplementary Fig. 2). Cocoa butter replacers (CBRs) are non-lauric fats with a fatty acid profile similar to cocoa butter, but a completely different triglyceride composition (e.g. PEE, SEE) Cocoa butter substitutes (CBSs) are lauric plant fats (containing lauric acid), chemically totally different to cocoa butter (e.g. major TGAs LLL, LLM, LMM), with some physical similarities; suitable only to substitute cocoa butter to 100% and often incompatible with CB^30^). Cocoa butter equivalents (CBEs) are non-lauric plant fats, which are relatively similar in their physical and chemical properties (e.g. major TGAs are POP, POS and SOS) to cocoa butter and can be proportionately mixed without affecting the properties of the cocoa butter. CBEs can be either cocoa butter extenders (CBEXs) which is a subgroup of CBEs not mixable in every ratio with cocoa butter or Cocoa butter improvers (CBIs) which have a higher solid triglyceride (SOS) content; used for improving soft cocoa butters^20^,^31^. Chocolate and confectionery industries give priority to fats which are rich in palmitic acid or stearic acid and are based on symmetric (POP-rich and SOS-rich) fats. Some research has shown that SOS rich fat confers a higher solid fat content which inhibits fat blooms and decreases the tempering time^26^. Therefore, SOS-rich fat could be used as a suitable raw material for the production of temperature-resistant hard butters in tropical countries^2^ and could also be used to improve the quality of soft cocoa butter^2^. Generally, lauric acid and hydrogenated fats are used to replace CB; these increase the levels of cholesterol whereas CBEs contain high oleic and stearic acids, which do not alter the levels of cholesterol in blood. Thus, CBEs represent a healthier and promising alternative to CB. CBEs used up to now are tropical SOS rich fat butters from species such as Shea (*Vitellaria paradoxa*), Kokum (*Garcinia indica*), Illipe (*Shorea stenoptera*), Mango (*Mangifera indica*) and Sal (*Shorea robusta*) butter and usually blended with palm (*Elaeis guineensis*) kernel oil stearin rich in POP^31^. Palm kernel oil consists of higher amounts of lauric acid, and relatively lower stearic and oleic acid than cocoa butter. The producing of CBA from palm oil needs intensive processing^8^. Palm kernel oil is used in preparing CBA as it is a very rich source of POP (51%). Sal butter is green in colour which limits its use in chocolate and confectionery products^13^. It is feasible to lighten butters but it is a very energy intensive procedure and costly, so industries prefer light coloured butters^11^. Kokum butter is grey coloured and mainly used as a CBE by blending with Mahua (*Madhuca longifolia*) and Phulwara (*Madhuca butyracea*) butter^32^. However, the extraction is only practiced at cottage scale and has no industrial application as yet^32^,^33^. Shea butter is known to have the highest unsaponifiable fat content (up to 10%) of any natural fat and the highest iodine value 52–56 (g Iodine/100 g fat) and is used as a cocoa butter substitute^34^ in the European chocolate and confectionery industry^35^. Illipe and mango butters can be used directly as CBE and Mango Butter (*Mangifera indica*) is comparatively good quality CBEs although the melting point (34–43 °C) is quite high^20^,^31^. There is therefore not enough reliable source of CBE available from natural fat sources^20^. Our study suggests that WMB is a potential high quality new CBE or improver as the fatty acid and triglyceride composition are very similar to CB as are the physical parameters.

However, going beyond an industrial utility, wild fruit is an important source of food, medicine and income for forest dwellers, tribal and marginalized rural people^36^. There are many wild fruits available in the forests that are underexploited. Moreover, information on their nutritional value and economic potential are unknown. Adding value to underutilized products through processing for products that have market value could generate a way to conserve those species and help to generate alternative income sources and reduce household poverty^37^. Additionally, collection and processing of these products can reduce household vulnerability to shocks and seasonal variations in other income sources^38^. For example, shea kernels from *Vitellaria paradoxa* are widely exported for use in the international cosmetic and chocolate industries. The annual value of total exports of shea kernels from Africa was estimated at USD 30 million in 2004^39^ and they represent one of Burkina Faso’s main export commodities^40^. Moreover, income from shea kernels has been shown to contribute as much as 12% of total household income for poor households and 7% of total household income for better-off households^40^. In Bangladesh, there are 47 edible wild forest fruits available^41^, an important one of which is the wild mango species (*M. sylvatica*). Wild mango is a multipurpose tree species used for multiple purposes, including edible fruits, pickles, fodder, fuelwood, vegetable, plywood, tea chest and match boxes^41^,^42^. A close genetic relationship between *M. indica* and *M. sylvatica* has been reported^43^,^44^ which indicates that *M. sylvatica* may have the potential to fulfil nutritional and livelihood needs. It is underutilized in Bangladesh as well as in other tropical countries due to a lack of awareness of it’s potential as a source of food and no established market demand^45^. However, this research confirms that this underutilized wild mango has the potential to be used as a unique source of cocoa butter alternative.

Bangladesh is one of the most densely populated countries in the world, with 2.14 million hectares forest area^46^. Millions of the poor and forest dwellers earn their livelihood from the forest^47^. Therefore, there is a socio-economic imperative to allow access for these forest dependent people to the natural resource. However, finding alternative income generating activities can secure income, improve livelihoods and conserve forest resources sustainably^48^. There is enormous potential for the development of a wild mango kernel based enterprise in Bangladesh as well as in other tropical countries for the production of wild mango butter. This will not only provide raw materials for the chocolate and confectionery industries but also offer opportunities to empower forest dependent people and small-scale farmers. There is therefore an urgent need to promote the domestication and commercial plantation of wild mango species to satisfy global and local demand for Cocoa Butter Alternatives. A recent study shows that it can be domesticated and introduced in small-scale forestry programs^15^. However, larger scale plantings will require field trials and an improved knowledge of the species silviculture. The current study may lead to the beginning of a domestication and commercialization of this wild under-utilized fruit species. However, more research on chocolate production using this butter and silviculture of this species is necessary to fully capture the value of this wild mango species. Additionally, collaboration between foresters, horticulturists, the food industry and policy makers is required to promote the domestication and commercialization of *M. sylvatica* fruits of Bangladesh and other tropical countries.

## Material and Methods

### Sample Collection, preparation, extraction and analysis

**Figure 1:**
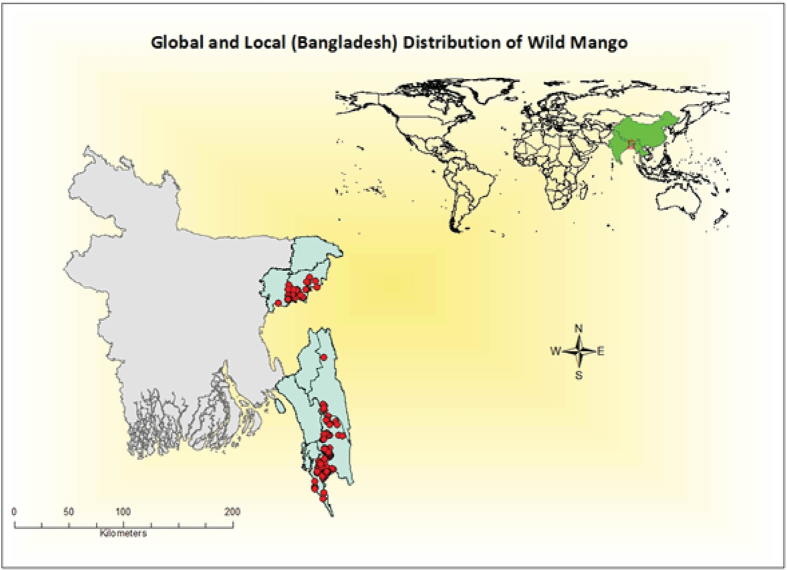
Global and Local Distribution of Wild Mango (*Mangifera sylvatica*). Spatial position of the site locations were plotted in global geo-political boundary available from Esri (http://www.arcgis.com/) and species presence locations were plotted in administrative map of Bangladesh using ArcGIS (version 10.3) software.

**Figure 2:**
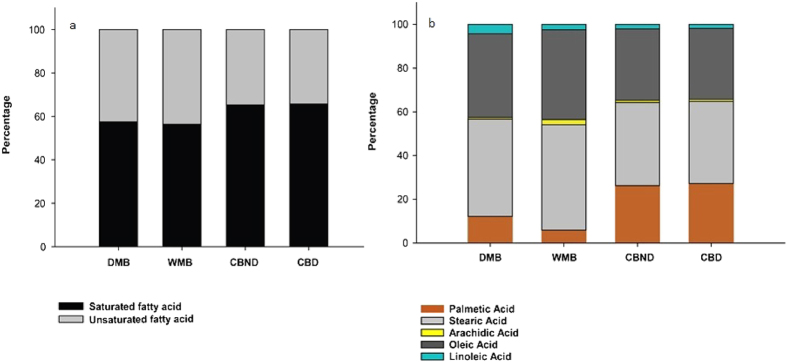
(**a**) Total saturated and unsaturated fatty acid content; Fig. 1. (**b**) Fatty acid profile of in WMB, DMB and CB (CBD and CBND).

**Figure 3:**
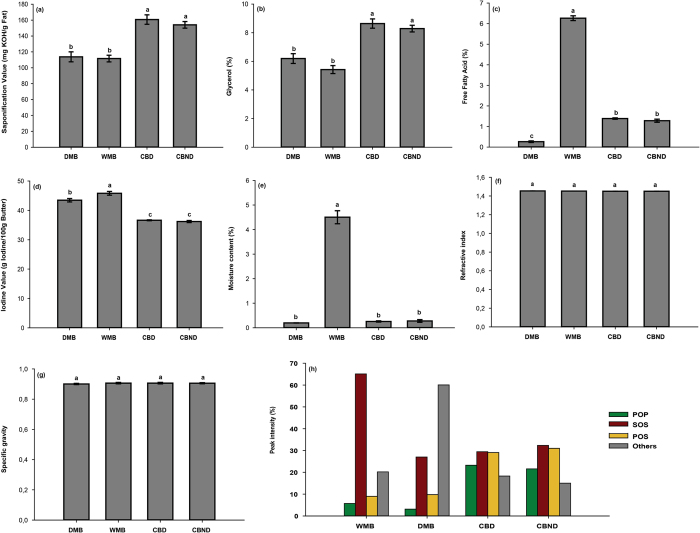
Physical properties of butter samples (**a**) saponification value (**b**) glycerol (**c**) free fatty acid (**d**) iodine value (**e**) moisture content; (**f**) refractive index; (**g**) specific gravity (**h**) major triglyceride percentage.

**Figure 4:**
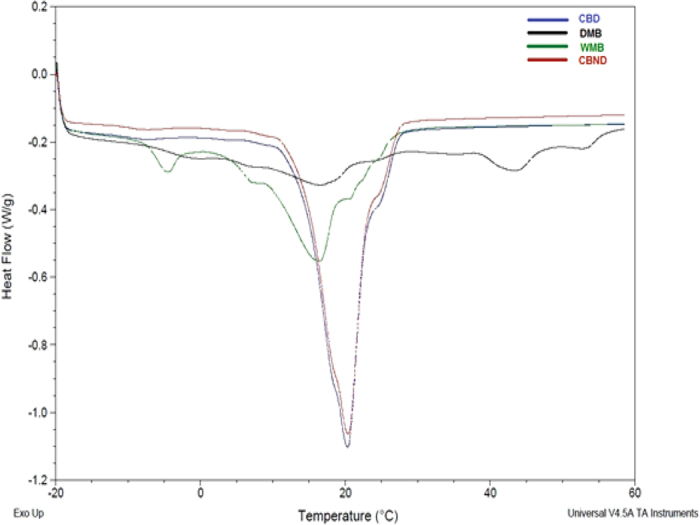
Melting profile of different butter using DSC.

**Figure 5:**
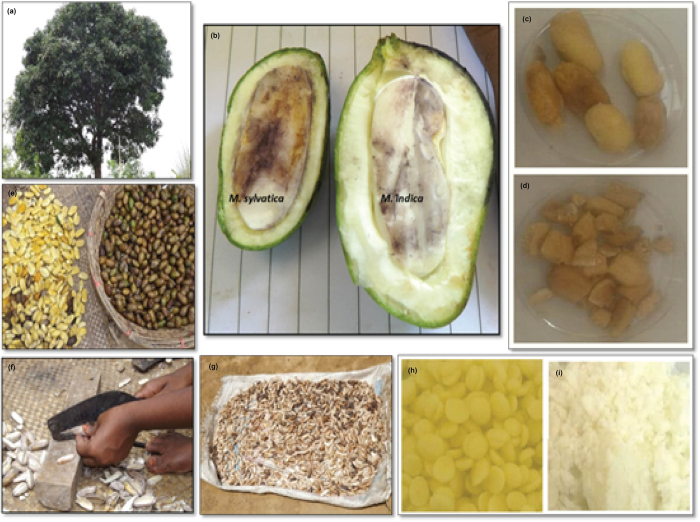
Collection, processing and preparation of wild mango butter from *M. sylvatica* (**a**) *Mangifera sylvatica* tree (**b**) fruit of *Mangifera sylvatica* with big kernel in left and fruit of *Mangifera indica* with big pulp in right (**c**) seed of *Mangifera sylvatica* (**d**) kernel of *Mangifera sylvatica* (**e**) *Mangifera sylvatica* seeds after retting (**f**) chopping the seeds (**g**) sun drying the kernels (**h**) cocoa butter (**i**) butter obtained from *Mangifera sylvatica* (Photo Credit goes to Sayma Akhter).

Mature fruits of *M. sylvatica* were collected from Cox’s Bazar, Bangladesh (Fig. 1), during April to June, 2014. After collection, fresh fruits were retted, de-pulped and sun-dried. The nuts were then separated from the kernel by using a hand betel nut cutter (Fig. 5). Phosphine fumigation was carried out before nuts were sent to Bangor University, UK for further processing. Mango butter was extracted using the SC-CO_2_ (Supercritical Carbon Dioxide Fluid Extraction) method. We obtained two cocoa butter samples from Callebaut chocolate industry (UK) and purchased 99% pure *Mangifera indica* butter (Domesticated Mango Butter, DMB) from the Soapery. Finally, analysed the physical (saponification value, iodine value, moisture content, specific gravity, refractive index, acid value, and glycerol percent), chemical (fatty acid profile and triglyceride composition) and thermal (melting profile) parameters of *Mangifera sylvatica* butter (Wild Mango Butter, WMB) and compared the results of WMB with the three other butter samples.

### Wild mango Butter extraction by SC-CO_2_

The wild mango kernels were extracted using Supercritical Fluid Extraction method^2^. A total 18.85 kg of dry ground mango kernel samples were loaded into the extraction vessel. The continuous methods of SC-CO_2_ extraction were carried out at pressures of 50 MPa, temperatures of 40 °C and at constant CO_2_ flow rate of 30 kg/hour. When pressurization initiated, the CO_2_ from the cylinder passed through the chiller at 0 °C and was pumped into the extraction vessel by a high-pressure pump. The fat was extracted from fat-rich CO_2_ by separators at one end of the instrument. Two separators were used though the entire process with the first separator being at fixed pressure and temperature of 80 MPa and 40° respectively. The second separator was maintained at room temperature and 55 MPa and desiccated the samples. CO_2_ was recirculated throughout the run time. Yield was calculated on a dry weight basis at the end of the process as g fat/kg mango kernel.

### Fatty acid and triglycerides profiling

The fatty acid composition of mango (*M. indica* and *M. sylvatica*) and cocoa butter (Deodorized and non-Deodorised) samples were done by GC (PerkinElmer Clarus 680)-MS (PerkinElmer Clarus 600 C). Five to seven mg of frozen butter dissolved in 1 ml of heptane and 0.05 ml of 1 N Methanolic NaOH were shaken at room temperature for two minutes. After 2 minutes when the two layers were separated the lower layer was discarded and the supernatant) used for GC-MS analysis. The analysis was done in triplicate. On the other hand, a direct infusion mass spectrometry method (API 150 EX MS System) used for the determination of Triglycerides. The nebulizer gas was N_2_. Scanning done for mass 100 to 1000 in an ESI Positive mode with a flow rate of 90 μl/min. Samples were run once for triglyceride profiling^2^,^7^ and percentage of triglycerides were calculated based on the peak intensity.

### Physio-chemical and thermal properties

Determination of saponification value, glycerol percentage, acid value, iodine value, moisture content, refractive index and specific gravity were carried out according to methods describes by^2^,^7^,^14^,^49^. Quantitative analyses were performed in triplicate and the results expressed as average ± standard deviation. One-way ANOVA was conducted to see any significance difference among four types of butter.Differential scanning calorimetry (DSC) used to monitor the melting profiles of the samples. A modified method of Yamoneka^29^ was used for this analysis. The method followed was heating- cooling - heating cycle. 1^st^ cycle (−20 °C to 60 °C) and 2^nd^ cycle (60 °C to −20 °C) was done to erase thermal memory and also to get rid of any unwanted materials. The final cycle (−20 °C to 60 °C) was recorded to get the melting profile of the samples. The heating rate was 10 °C/min and cooling rate was 2 °C/in.

## Additional Information

**How to cite this article**: Akhter, S. *et al.* *Mangifera sylvatica* (Wild Mango): A new Cocoa butter alternative. *Sci. Rep.* **6**, 32050; doi: 10.1038/srep32050 (2016).

## Supplementary Material

Supplementary Information

## Figures and Tables

**Table 1:** Triglyceride Profile *of M. sylvatica* butter, *M. indica* butter and Cocoa Butter.

Triglycerides	Triglycerides	WMB	DMB	CBD	CBND
1,3-dipalmitoyl-2-oleoylglycerol	POP	√	√	√	√
1-palmitoyl-2-oleoyl-3-stearoyl-glycerol	POS	√	√	√	√
1,3-distearoyl-2-oleoyl-glycerol	SOS	√	√	√	√
Trioleoyl-glycerol	OOO	√		√	
1-Arachidoyl-2-Oleoyl-3 Linoleyolglycerol	AOLo	√			√
1,2 -Palmitoyl-3-Linoleoylglycerol	PPLo			√	√
1-stearoyl-2,3-dioleoyl-glycerol	SOO		√		
1-stearoyl-2-oleoyl-3-Arachidoyl-glycerol	SOA		√		

**Table 2:** Melting Characteristics of different butter samples.

Sample	T_onset_(°C)	T_offset_(°C)	T_peak_(°C)	Enthalpy change (J/g)
*Mangifera sylvatica* butter	−7.20	25.88	16.18	16.3383
*Mangifera indica* butter	−5.36	58.00	16.66	10.4353
Cocoa Butter Deodorized	14.64	26.99	20.27	42.1099
Cocoa Butter Non-Deodorized	14.58	26.99	20.34	45.7108
