# Supplementary material for: Mangifera sylvatica (Wild Mango): A new cocoa butter alternative
Source: Sci Rep. 2016 Aug 24;6:32050. doi: 10.1038/srep32050 (PMC4995435; doi:10.1038/srep32050)
Supplement: Supplementary Information [file srep32050-s1.pdf]

Mangifera sylvatica (Wild Mango): A new cocoa butter alternative

Sayma Akhter[1*], Morag McDonald[1] & Ray Mariott[2]

[1]School of Environment, Natural Resources and Geography, Bangor University, Gwynedd, LL57 2UW, UK (Corresponding author: azs007@bangor.ac.uk/ sayma_sust@yahoo.com)
[2]Biocomposites Centre, Bangor University, Gwynedd LL57 2UW, UK

Supplementary materials

Supplementary Table 1: Fatty acid content of different butters

**Species**	**Butter**	**Saturated fatty acid (%)**	**Unsaturated fatty acid (%)**
Mangifera sylvatica	Wild mango butter (WMB)	56,44[a]	43,56[a]
Mangifera indica	Domesticated mango butter (DMB)	57,51[a]	42,49[a]
Theobroma cacao	Cocoa butter deodorized (CBD)	65,78[b]	34,22[b]
Theobroma cacao	Cocoa butter non deodorized (CBND)	65,30[b]	34,70[b]

Different letters represent significant differences ($P< 0.05$)

Supplementary Table 2: Fatty acid profile of different butters

Butter	Saturated fatty acid (%)			Unsaturated fatty acid (%)	
	**Palmitic Acid (16:0)**	**Stearic Acid (18:0)**	**Arachidic Acid (20:0)**	**Oleic Acid (18:1)**	**Linoleic Acid (18:2)**
DMB	12,18[b]	44,53[b]	0,80[c]	38,18[b]	4,31[a]
WMB	5,86[c]	48,23[a]	2,34[a]	41,12[a]	2,45[b]
CBD	27,24[a]	37,53[c]	1,01[b]	32,42[c]	1,80[b]
CBND	26,25[a]	38,02[c]	1,03[b]	32,66[c]	2,04[b]

Different letters represent significant differences ($P< 0.05$)

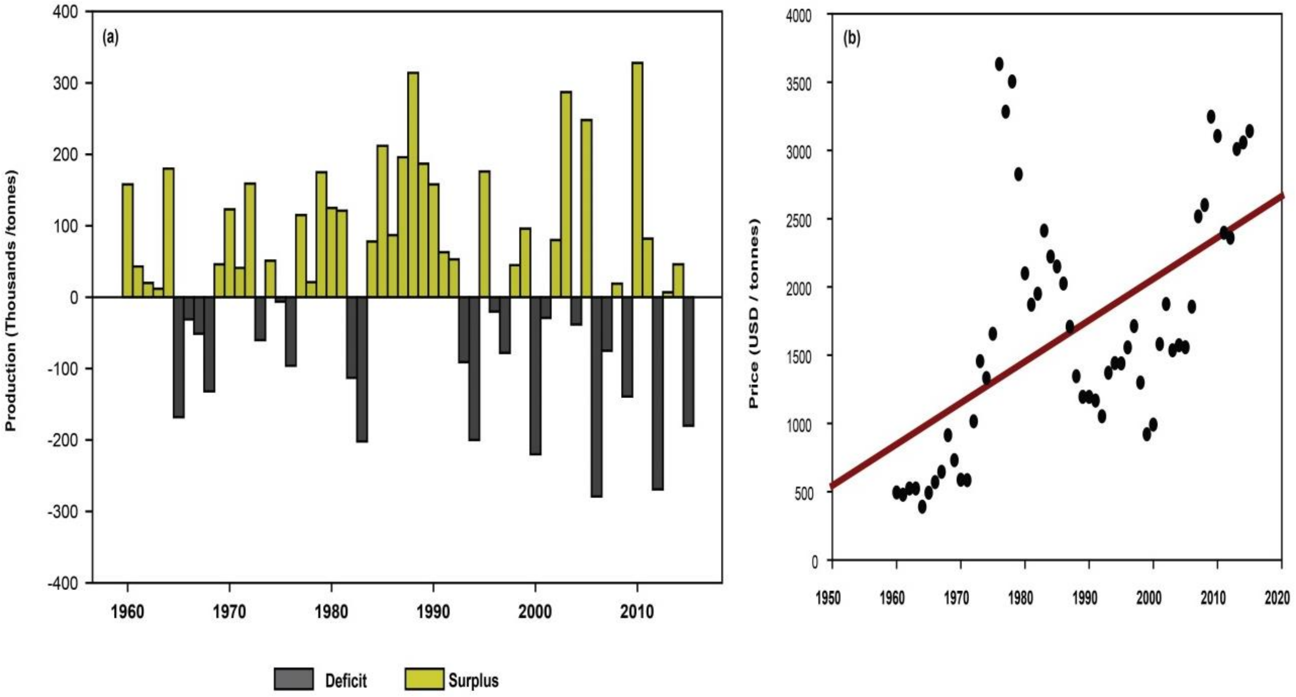

Supplementary Figure 1: Cocoa butter production and price from 1960 to 2015 (ICCO 2016a; ICCO 2016b)

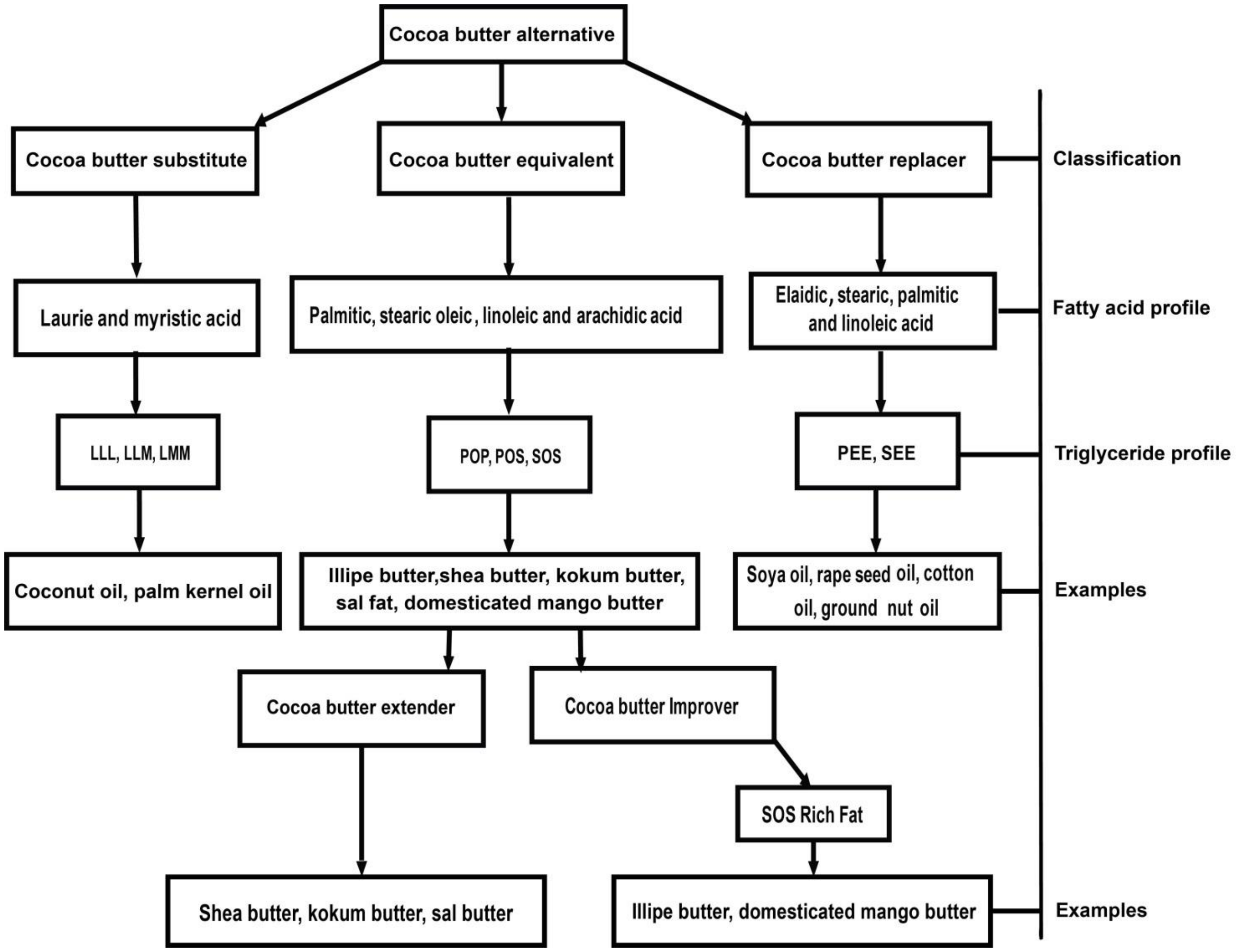

Supplementary Figure 2: Cocoa butter alternative classification (adapted and modified from Lipp and Anklam 1998)

Supplementary references

ICCO 2016a. ICCO Quarterly Bulletin of Cocoa Statistics, Vol. XLII, No. 1, Cocoa year 2015/16Published: 26-02-2016. Available at http://www.icco.org/about-us/international-cocoa-agreements/cat_view/30-related-documents/47-statistics-supply-demand.html

ICCO 2016b. ICCO Monthly Averages of Daily Prices. Available at http://www.icco.org/statistics/cocoa-prices/monthly-averages.html

Lipp, M. & Anklam E. Review on cocoa butter and alternatives for use in chocolate. Part A: Compositional data. *J. Food Chem*. **62** (1), 73-97 (1998).
